# Case of Constricted Vagina and Occlusion of the Os Uteri

**Published:** 1851-11

**Authors:** Laurence Turnbull

**Affiliations:** Locust street, Philadelphia


### *Case of Constricted Vagina and Occlusion of the Os Uteri.* By LAURENCE TURNBULL, M. D.

Mrs. G., æt 39, wife of an estimable farmer, residing at Livonia, New York, came under my care for surgical treatment on the 14th of May, 1851, when the attending physician Dr. J., Clark, related to me the following narrative of the case.

She had passed through most of the exanthematous diseases, suffered for many years with a large thyrocele that eventually disappeared under the use of iodine, and since her eighteenth year had seldom escaped a daily headache accompanied with nausea and frequent vomiting.

After two successful confinements she was seized in labour, on the 15th of April, 1848, at 2 P. M., having been pregnant from the best estimation, 300 days. The labour continued twenty-four hours, and not the least advancement of the head could be perceived; it lay in the greater basin of the pelvis, hard, unyielding, and apparently much too large for engagement. A consultation had been requested and obtained, when exhaustion coming on, it was resolved to perforate the head, which being done, the occiput soon presented itself at the vulva, and shortly afterwards the body of the child was drawn away, requiring, however, considerable mechanical force.

Upon examination, the child presented many evidences of unusual development; the scalp was abundantly supplied with hair, and ossification of the cranial bones had proceeded so far as to prevent that moulding of them which facilitates parturition, making it very difficult to perform craniotomy.

Inflammation of the pelvic viscera supervened, accompanied with severe fever, and very fetid and scanty lochial discharge; after a protracted illness of six weeks, she recovered sufficiently to engage in her domestic affairs, at which time a sanguineous discharge took place from the vagina. Other than that, she never evacuated any menstrual fluid since her confinement; her catamenia never had any exact periodicity, or fixed quantity, sometimes occurring once in three weeks, at others prolonged; from motives of fastidious delicacy, no vaginal examination was made during her sickness.

She spent the two succeeding years with some degree of comfort, experiencing, however, much difficulty in walking, from a sense of constriction across the anterior portion of the pelvis; her headache and vomiting were exacerbated at irregular intervals; the latter occurred soon after rising in the morning, ejecting a fluid of blue, green, or yellow color, often tinged or mingled with blood.

On the 16th of March, 1850, frequent and difficult micturition appeared, together with pains similar to the expulsive ones of labour originating in the axis of the pelvis, and radiating to the sacrum, lumbar regions and thighs: immediately subsequent to this the whole abdomen became excessively sensitive and tympa. nitic with high fever, great prostration and pulse ranging from 120 to 150 per minute; these symptoms subsided under the free administration of quinia, tinct. opii, and revellents to the abdomen.

An examination by the rectum at this time, gave nothing but the sensation of a corrugated condensed tissue in front of the intestine; the vagina was constricted to an orifice one line in diameter, situated at the junction of the right rami of the ossa ischii and pubis ; the orifice was at the bottom of a funnel-shaped depression, three-fourths of an inch in depth, and compressed on the right, where it was contiguous to the bone ; by using considerable force, and spending several hours in the attempt, the blunt end of a common pocket probe was insinuated full five inches, taking a direction to the right of the promontory of the sacrum ; female catheters of a graduated size, were afterwards introduced, and at a still later period, some silver gilt vaginal bougies manufactured by Mr. Warner of this city, and recommended by Prof. C. D. Meigs, who on many occasions kindly advised as to the treatment of the case.

1850, September 14th. By using the bougies the vagina has been nearly restored to its natural dimensions for the distance of three inches from the vulva : (she was unable to wear them constantly and they were therefore applied once a day, or as often as the state of the parts would admit ;) at this point is a circular constricting band of a hard and fibrous character, through the centre of which, a common catheter passes freely, and rotates with ease in different directions, giving the impression that there is a free space beyond it ; the opening sometimes admits a bougie three-fourths of an inch in diameter, at others the catheter cannot enter. Within the vulva to the left, and partially in front of the vagina, could be felt an oblong, oval, smooth body, visibly occupying all that side of the excavation, occasionally soft, compressible and almost disappearing ; again it contracts, bears down upon, and pushes the finger to the right, when introduced into the vagina ; this would seem to account for the direction of the probe when first inserted. All attempts to overcome the constriction by dilatation gave immediate relief from the singular sensation in the anterior portion of the pelvis ; the instruments when removed were covered with a clear sanguinolent mucus.

By palpation of the bladder when distended, it appears to occupy its natural position, but above the umbilicus can be felt the uterus, five inches or more in diameter at the summit, hard and condensed when the pains come on, soft when they are absent; at first occupying the right lumbar region, it now rests in the mesial line of the body ; by ballottement it evidently has a connexion with the tumor in front of, and to the left of the vagina, one being pushed up the other follows it, and vice versa: fluctuation is perceptible in both.

Tenderness of the abdomen has entirely subsided, but expulsive tenesmic pains recur at regular intervals of three or four weeks, commencing at 7 A. M., disappearing about 12 M., and continuing from one week to three.

At these periods she has no fever, though the circulation is somewhat accelerated, the extremities become cold, and the vomiting is increased, large quantities of muco-gastric fluid being ejected: her gums in the morning are encrusted with a layer of sanguineous sordes, sufficiently thick and tenacious to be removed by the finger, which as it is not the result of scorbutus oris is an anomalous perversion of function. During these attacks she is generally confined to bed with loss of appetite and constipation, though retaining thus far her usual fulness of body; she obviates the constipation by confectio sennæ, and partially allays her pains with tinct. opii.

Sept. 20th, 1850. An important change occurred; the tumor was reduced to nearly one half its former size, attended with severe pain, constitutional prostration, increased emesis, the evacuation of a large quantity of pseudo-membranous substance, from the intestine, also, some pinkish fluid from the vagina; after this, her pains ceased for some time, appetite improved, and she was able to go about the house.

1851, March 8th. The resisting constriction had been broken up, but no os tincæ was discernable by the touch beyond it, and the pain had commenced recurring as before, requiring large doses of laudanum to subdue them; the operation of puncture was proposed by the attending physician, but Professor Meigs considered it a dernier resort, and advised the employment of a sponge tent, which proved exceedingly serviceable in distending the vagina, and making the subsequent operation more easy of performance.

1851, April 21st. The increasing distension of the uterus had caused it to descend and fill the excavation, compress and shorten the vagina, as well as render defecation and micturition very difficult. She was much exhausted, her pains becoming insupportable, even under the daily consumption of f.℥ss. tinct. opii. Life seemed endangered, and some prompt action was requested, and application was therefore made to several medical gentlemen of this city to visit her; I complied with the request, and arrived there on the 14th of May.

Upon the occasion of my visit, she was in bed, suffering little or no pain; her countenance, cheerful, pallid and emaciated, was devoid of any cachectic hue; her nails were aduncated, like those of tuberculous patients, and she occasionally had cough, yet no evidences of pulmonary disease were afforded by physical diagnosis.

The tumor, occupying the umbilical and hypogastric regions, now measured eight inches from its summit to the symphysis pubis, and six inches at its largest diameter; the mucous lining of the vagina appeared healthy, save a small abrasion at its distant extremity; its lateral dimensions were natural; it would, however, admit the speculum only two and seven eighths inches, through which, at the bottom of the canal, were seen two semilunar elevations, three-fourths of an inch long, their long diameter being antero-posterior, and separate from each other about one half of an inch: the intervening space was occupied by a dense and fibrous substance, against which probes were pressed on many points, but no place was found where they would enter; this, on consultation, we determined to make the point of puncture. About an ounce of urine could be retained at once, the catheter, when introduced, taking a direction in front of the tumor, which still remained on the left of the vagina, and could be felt through the rectum.

The bladder and rectum being well evacuated, and a bandage applied, on the succeeding day, the 15th, in company with Dr. Clark and his son, Dr. H. Clark of Philadelphia, and the husband and father of the lady, I thrust in an exploring trocar at the selected place; its entering extremity moved freely in different directions, and some blackish fluid escaped on its removal, when a curved trocar and canula the size of a crow's quill, were next introduced, passing through a resisting tissue one half of an inch in thickness.

On withdrawing the trocar, a thick, ropy, molasses-like, deep hyacynth-red fluid flowed slowly away, and continued to escape, until twenty-seven ounces were collected, the canula being retained in situ for the two following days, during which time much more was evacuated; the tumor in the abdomen contracted to the size of the uterus after delivery; the one on the left of the vagina disappeared, and that canal elongated itself to nearly its normal extent; she soon became more lively, moved herself with alacrity, partook of supper, and that night enjoyed the most refreshing sleep she had known for many months.

The 18th was about the period of the recurrence of pain, and an interesting chain of phenomena presented themselves; head-ache, vomiting of a green liquid, hurried and oppressed respiration, sibilant ronchus, exquisite sensibility of the hypogastric region, pulse 130, full and soft, the uterus increasing in size, but without an antecedent chill; calomel, opium and neutral mixture, with sinapisms, and opiate cataplasms to the abdomen, were ordered, and at evening the symptoms had lessened in intensity, to reappear on the next day, but with diminished severity.

During this time the puncture had been kept patulous by the occasional introduction of a Simpson's sound, the end of which moved freely as if in a large quantity of fluid, but none made its escape after taking it away.

May 20th.—This morning she appeared quite at ease, though still in bed. Left her, on my departure for Philadelphia, in the hands of Dr. Clark, who has kindly furnished the following notes:—

"21st.—Fell into syncope at 10, A. M., but soon revived; tenderness of the abdomen diminished; appetite improved and sleeps well; quinia and brandy administered freely, and Tr. Opii M. xxv. given occasionally.

"22d.—Pulse 100—this rate has become almost habitual; no pain, and feels vigorous; sound passed with no resistance, but after taking it away a large quantity of fluid escaped of less consistence than the former, with an intolerable odor of sulphuretted hydrogen.

"23d.—Fluid commenced flowing per se at 3, P. M., having ceased during the preceding night; abdomen flaccid without soreness, and a marked sensation of relief in both iliac regions; occasional colicky pains.

"24th.—Four napkins saturated with the fluid, alternately consistent and limpid, but retaining the nauseous odor; she is loud in her exclamations of relief; enjoys the free expansion of her lungs and is full of vivacity. Quinia and brandy still continued, with Tr. Opii, p. r. n.

24th, Evening.—Discharge continued without interval: less odor; soreness in the region of uterus and right ovarium; tumor in the right iliac region; pulse 92, tongue coated, bowels free, little perspiration, no fever, appetite indifferent; Tinct. Opii., M. xxv., night and morning.

25th. Two attacks of emesis after leaving her yesterday; vagina syringed with a solution of castile soap and mucilage: large discharge with occasional grumous clots of very dark color and offensive odor yesterday afternoon; none since 6 P. M. yesterday; tumor in right iliac region the size of a goose egg and very sensitive; pulse 100 appetite variable; slept well the preceding night.

26th, 11 A. M. Free evacuation from the bowels accompanied with pain; refreshing sleep last night: free discharge from the uterus of a blackish color resembling the dregs on a laudanaum filter, and bad odor: increased fulness and tenderness in the hypogastric and iliac regions; pulse 90; copious perspiration; appetite bad. Treatment: Quinia gr. ss., every two hours; Brandy and Tinct. Opii. occasionally; ointments of Sugar of Lead and Opium locally.

28th. Improved in all respects; pulse 88 and soft; tongue nearly clean, slept well last night; perspiration free, appetite improved, feels more vigorous; bowels free and discharge continues. Treatment same as before.

28th, 5 P. M. Passed the day quietly; three evacuations from the bowels attended with pain: vaginal discharges of less odor and consistence; diminished abdominal tenderness; appetite the same; pulse from 80 to 90.

29th. Patient expressed herself better than any day since the operation; tumor smaller; tenderness and tension of the abdomen subsided; emplast. belladonnæ to the hypogastric region; pulse 90: strength, appetite, &c., improving, while the disharge continues with odor like that of an accouchée. M. xxv. Tinct. Opii.

30th. No change from yesterday.

June 2d. Patient sent for me: was very weak: pulse 132, the previous night a restless one; severe pain in both iliac regions, not relieved until she had taken Tinct. Opii. f℥ii; discharge watery and offensive: vomiting recurred; bowels confined, tongue coated, no appetite and thirsty. Quiniæ Sulph. gr. ss. every three hours. Brandy occasionally.

3d. 5 P. M. Requested to visit the patient at 1 A. M. Very feeble, pulse scarcely perceptible: Quinia gr. ss. every hour; brandy liberally: chickenbroth tablespoonful every half hour: at this time much more comfortable, pulse 120, bowels confined, urine high colored, perspiration copious, less tenderness and occasional vomiting. Opiate cataplasms to the abdomen; sound passed readily: after its removal two three ounces of a cineritious colored fluid flowed away: it was extremely offensive; administered two injections of f℥iv. each of tepid water; gas escaped from the uterus with a hissing noise followed soon by a loud report, then fluid with a gurgling sound having the appearance of soap suds.

4th, 4 P. M. Rested well last night, no pain or soreness since the injection; a stool after three enemata; urine natural and freely evacuated: nothing from the uterus since the washing out; pulse 110, full and soft; breathing natural, perspiration free, tongue clean at tip and edges, no appetite, some thirst, stronger, vomited several times; abdomen tympanitic, relieved by enemata of vinegar and water.

June 5th, 6 P. M. Saw the patient this morning; passed the night pleasantly; pulse 100, perspiration less profuse, abdomen less tympanitic and less tender, bowels confined. Prescribed Hyd. Chlorid. Mit., gr. iv, enemata if requisite, and alcoholic lotions; she has been quiet through the day, vomited several times fluid of a green color: two enemata administered followed by two natural evacuations; no discharge from the uterus; urine of natural appearance and quantity, pulse 120, white incrustation on the roof of the mouth; os of healthy appearance, bile just escaped per anum; Quinia gr. ss. every hour, brandy occasionally; Tinct. Opii. gt. xxv, twice a day.

6th. Little sleep last night; frequent vomiting of green fluid; changed this morning to brown color similar to that thrown off at previous times; countenance haggard, apthous incrustation of the palate and fauces; membrane beneath of a dark mahogany hue; pulse small and frequent; extremities cold, inclination to profuse perspiration; bowels slightly opened, healthy bile evacuated; urine passes freely and of natural color; tongue less coated, no appetite or pain; less tumefaction of the abdomen; slight tenderness in the left iliac region; no discharge from the uterus, and no odor in the room. Decoction of tansy to the abdomen for the last twenty-four hours; the pain, a stimulus to which she has been accustomed, being gone, nothing appears adequate to the task of rallying the worn out energies of the system; 3 P. M., these notes close with the patient's life; I have no remarks to make other, than that I wish the operation had been performed six months earlier."

I regret exceedingly that Dr. Clark made no post-mortem examination, but the practical deductions to be drawn from the case are the following:

That, in all cases of instrumental delivery where sloughing occurs, great care should be exercised in keeping the parts dilated, by means of bougies, and daily examinations should take place, so as to break up irregular contractions. In this case, nearly two years were allowed to elapse, and the menstrual fluid accumulated; the watery particles passing away, there was left a concentrated fluid, a portion of which may have escaped into the cavity of the peritoneum, through the fallopian tubes, there causing acute inflammation, which is probable from the symptoms, on the 16th of March, 1850.

It has been found by the most recent observers, that when the womb is dilated by fluid within its cavity, the fallopian tubes are patulous, and therefore, when severe contractions occurred, a portion of the fluid might readily escape in that way.

In most cases where this operation has been recorded, it was performed soon after the accumulation occurred, and not left until the powers of the system were weakened by long continued suffering, and the patient unable to perform the functions so important to health.

*Locust street, Philadelphia, October,* 1851.

